# Investigation of the underlying factor structures of social interaction impairments in children diagnosed with autism spectrum disorder at autism centers, Addis Ababa, Ethiopia: principal component analysis of the autism treatment evaluation checklists

**DOI:** 10.11604/pamj.2025.51.81.47147

**Published:** 2025-07-28

**Authors:** Worku Abie Liyew, Ayalew Moges, Fikirte Girma, Workeabeba Abebe, Mekbeb Afework

**Affiliations:** 1 Department of Anatomy, School of Medicine, College of Health Sciences, Addis Ababa University, Addis Ababa, Ethiopia,; 2 Department of Biomedical Sciences, School of Medicine, Debre Markos University, Debre Markos, Ethiopia,; 3 Department of Pediatrics and Child Health, School of Medicine, College of Health Sciences, Addis Ababa University, Addis Ababa, Ethiopia,; 4 Department of Psychiatry, School of Medicine, College of Health Sciences, Addis Ababa University, Addis Ababa, Ethiopia

**Keywords:** Autism, factor structure, social interaction impairments, principal component analysis

## Abstract

**Introduction:**

social interaction impairments are the main feature of individuals diagnosed with autism spectrum disorder (ASD), making it a significant area of concern for both the children affected and their families. This study aimed to investigate the underlying factor structures of social interaction impairments in children diagnosed with autism spectrum disorder at autism centers in Addis Ababa, Ethiopia.

**Methods:**

a cross-sectional study was conducted among 145 children diagnosed with autism spectrum disorder. Various traits of social interaction impairments associated with autism were measured using the Autism Treatment Evaluation Checklist. A principal component analysis (PCA) employing a varimax rotation was used to identify the underlying factor structures of social interaction impairments.

**Results:**

six-factor structures of social interaction impairments were identified: challenges in interpersonal relationships and affection (eigenvalue: 4.99, variance: 13.437%), emotional insensitivity (eigenvalue: 1.714, variance: 11.837), poor gesture and attention (eigenvalue: 1.648, variance: 10.652), social withdrawal (eigenvalue: 1.40, variance: 8.932), behavioral resistance (eigenvalue: 1.270, variance: 8.011), and limited sharing (eigenvalue: 1.044, variance: 6.771). These factors accounted for approximately 60% of the total variance.

**Conclusion:**

the social interaction impairments in autism are multidimensional, presented in six distinct patterns. It is important to consider these patterns when supporting children with autism spectrum disorder.

## Introduction

ASD is a complex and heterogeneous neurodevelopmental disorder primarily affecting brain development and functioning [1-4]. It is characterized by impairments in two domains of functioning: (1) reciprocal social interaction and language communication, and (2) restricted, repetitive, stereotyped interests and behaviors [5]. Among the most significant challenges that people with ASD experience are impairments in social functioning and difficulty understanding the social world and maintaining relationships in community settings, including home and school [6]. This social difficulty manifests in a range of behaviors, including challenges in recognizing social signals, difficulty in forming peer relationships, lack of understanding of social norms and emotional responses, and limitations in expression and gesture [7]. Children with autism often struggle to initiate or sustain conversations, engage in imaginative play, or interpret non-verbal communication such as facial expressions and body language [8]. These difficulties may lead to social isolation, loneliness, and frustration, which can, in turn, exacerbate other behavioral issues such as anxiety, depression, and aggression [9,10]. Moreover, these social challenges can also interfere with academic performance for learning in the school environment [11].

Social interaction impairments not only affect the children but also impact family life as a whole. The emotional and physical demands for caring profoundly alter family interpersonal relationships, creating challenges to understand and manage [12]. They also develop stress, uncertainty, and future frustration on how to manage their children in the social environment; parents feel isolated due to their child's challenges in interacting with others [13]. Siblings may experience emotional distress, and the strain can lead to burnout, affecting parents' mental health and relationships [14]. These impairments also have a societal impact, as children with autism may struggle to form friendships and participate in community activities, leading to exclusion and stigmatization.

In Ethiopia, currently, there is no exact official data on the prevalence of autism; however, according to the estimation made by Nia Foundation, 500,000 children living with autism and related developmental disorders were estimated in 2002, and by 2015, this number had increased to about 530,000 [15]. The rising prevalence of autism highlights the importance of increasing our understanding of social interaction impairments, which significantly impact the quality of life for individuals with autism. To the best of our knowledge, the underlying patterns of social interaction impairments have not yet been studied in Ethiopia. The limited understanding may hinder the development of effective support strategies, as their origins and manifestations are complex [16]. Furthermore, much of the existing understanding of autism comes from high-income countries [17,18], which may not be directly applicable to the Ethiopian context due to cultural, environmental, and socioeconomic differences [19]. In Ethiopia, while autism centers in Addis Ababa provide intervention and support services, no study has tried to investigate the social interaction components of children diagnosed with ASD.

By PCA on autism treatment evaluation checklists (ATEC) [20], the current study investigated the underlying factor structures of social interaction impairments in children diagnosed with ASD at autism centers in Addis Ababa. The findings of this study could contribute to informing concerned bodies about the rich and complex nature of factor structures in the sociability domain of autism at autism centers in Addis Ababa and guiding them to develop culturally relevant and targeted interventions that can improve the quality of life for children and their families.

## Methods

**Study setting, design, and period:** the study was conducted at two autism centers in Addis Ababa: Nehemia Autism Center and Nia Foundation. A cross-sectional study was carried out among children diagnosed with ASD. Data collection was carried out from June 2024 to October 2024.

**Inclusion and exclusion criteria:** all children diagnosed with ASD between the ages of 4 and 16 were the source population. The study population consisted of individuals who met the eligibility criteria. The study excluded participants who did not have a clinical diagnosis of ASD, caregivers, or parents who were unwilling to provide consent. Additionally, participants who were not present during the data collection period and those with comorbidities, such as seizures or those taking medication, were also excluded from the study.

**Sample size determination and sampling technique:** sample size was determined by considering the ratio of study variables for this study (p=20) to the number of study subjects (N) in principal component and exploratory factorial analysis [21], and the sample size was calculated to be 145. Consecutive sampling was conducted based on the presence of autistic children in the classroom.

**Data collection:** sociodemographic data and various aspects of social interaction traits were collected through a pretested structured questionnaire administered by interviewers, with responses provided by the caregivers of the study participants. Autism traits of social interaction were measured using the validated Amharic version of ATE [22]. This questionnaire tool is used to measure clinical improvements and symptom severity for ASD [23-26] and has been validated through various longitudinal studies and clinical trials [22,24,27-32]. ATEC evaluates the improvement of autistic behaviors across four domains: I) speech/language/communication (14 items, score range from 0 to 28); II) sociability (20 items, score range from 0 to 40); III) sensory/cognitive awareness (18 items, score range from 0 to 36); and IV) health/physical (25 items, score range from 0 to 75). In the present study, the sociability domain was examined to identify the different patterns of social interaction impairments and group them into distinct categories. Each item was scored using a 0-2-point scale derived from responses of severity answered on the ATEC form marked as not descriptive=0, somewhat descriptive=1, and very descriptive=2.

**Data analysis:** it was conducted by SPSS Version 22 Statistical Software. The underlying factor structures of impairments in social functioning were performed using PCA and varimax rotation. The suitability of the data for factorial analysis was tested by the Kaiser-Meyer-Olkin (KMO) measure of sampling adequacy and Bartlett's test of sphericity, and a KMO value greater than 0.6 was adequate for analysis [25]. The numbers of principal components and factors to be retained were determined by examination of the eigenvalues and scree plot. Eigenvalues greater than 1 and the “elbow,” where the eigenvalues start to level off, were used [33]. The variable composition of each factor was examined by the analysis of factor loading on the rotated component matrix. High variable loadings, above 0.3, were considered for each factor [34]. For items exhibiting multiple cross-loadings, the item with the highest loading on a particular factor was selected for inclusion in the final model [35]. The variables in each factor were examined and labeled based on their common meanings. To describe each factor, simple terms were used [33].

**Ethical consideration and consent to participate:** ethical approval was obtained from the Institutional Review Board of the College of Health Sciences, Addis Ababa University, and a protocol number was provided: 097/23/Anat. Written informed consent was obtained from the caregivers of each child participating in the study.

## Results

**Sociodemographic characteristics:** a total of 145 children diagnosed with ASD were enrolled in this study. Their mean age was 9.2 (SD=3.1), and the majority of the study participants were males (83.4%) (Table 1).

**Table 1:** sociodemographic characteristics of study participants and their parents (n=145)

Sociodemographic variables		Frequency	Percent
Sex of children	Male	121	83.4
	Female	24	16.6
Educational status (for parents)	Not write and read	5	3.4
	Primary school	4	2.8
	Secondary school	49	33.8
	Diploma	34	23.4
	Degree and above	53	33.6
Marital status parents	Married	106	73.1
	Single	39	26.9
Occupation of parents	No work	55	37.9
	Private	56	38.6
	Government employ	28	19.3
	Merchant	5	3.4
	Farmer	0	0
	Daily laborer	1	0.7
Mean ages of children=9.19(SD=3.1)			
Median ages of children=8			

**Statistical descriptions of items in the sociability domain:** the means for each item related to the sociability of the study participants ranged from 0.39 (SD=.766) to 1.22 (SD=0.979). The overall weighted average of the mean was approximately 0.79 (Table 2).

**Table 2:** percent, means, and standard deviations of each item assessed in the sociability domain (n=145)

Items	Frequency in %			Mean	Std. Deviation
	0	1	2		
Not sociable/approachable	53.8	11.7	34.5	.81	.923
Ignore other people	34.5	9	56.6	1.22	.931
Pay little or no attention when addressed	54.5	10.3	35.2	.81	.930
Uncooperative and resistant	45.5	11.7	42.8	.97	.942
No eye contact	55.2	16.6	28.3	.73	.876
Prefer to be left alone	42.8	15.2	42.1	.99	.924
Show no affection	57.2	4.8	37.9	.81	.960
Fails to greet parents	54.5	5.5	40	.86	.965
Avoid contact with others	48.3	6.9	44.8	.97	.968
Does not imitate	58.6	9.7	31.7	.73	.915
Dislikes being held/cuddled	77.9	4.8	17.2	.39	.766
Do not share or show	56.6	8.3	35.2	.79	.937
Do not wave bye-bye	53.8	7.6	38.6	.85	.953
Disagreeable/not compliant	53.1	21.4	25.5	.72	.846
Temper tantrums	42.1	10.3	47.6	1.06	.949
Lacks friends/companions	37.2	9	53.8	1.17	.943
Does not smile	57.2	5.5	37.2	.80	.955
Insensitive to others' feelings	50.3	9	40.7	.90	.953
Indifferent to being liked	53.1	4.8	42.1	.89	.973
Indifferent if parent(s) leave	46.2	4.8	49	1.03	.979

Scores range from 0: not descriptive, 1: somewhat descriptive, 2: very descriptive

**Sample adequacy tests and correlation analysis:** the KMO value was 0.752, which was higher than the required minimum level (i.e., the data was suitable for factorial analysis). This was also supported by the Bartlett chi-squared test, 842.592, which was statistically significant (p=000) (Table 3). Thus, the null hypothesis that the items do not correlate was rejected.

**Table 3:** Kaiser-Meyer-Olkin and Bartlett's test

Kaiser-Meyer-Olkin measure of sampling adequacy		752
Bartlett's test of sphericity	Approx. Chi-Square	842.596
	Df	190
	Sig	000

**Figure 1:**
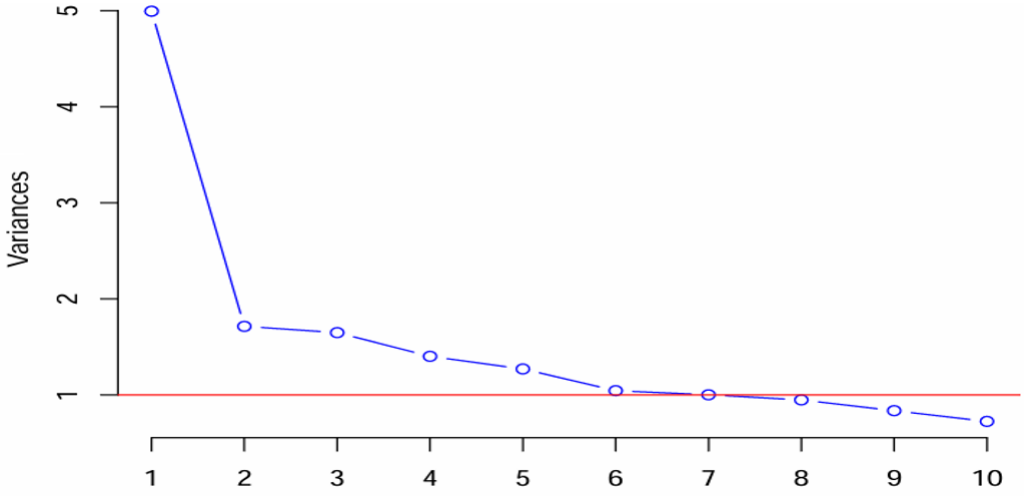
scree plot illustrating the variances associated with each principal component analysis

**Principal component analysis:** an examination of eigenvalues and scree plot indicated six PCA to be retained, and they accounted for 60% of the total variance. Before rotation, the eigenvalues and percent of variance for these six components were 5.0 (24.98%), 1.7 (8.57%), 1.6 (8.24%), 1.4 (7.05%), 1.3 (6.34%), 1.0 (5.21%), respectively (Table 4 and Figure 1).

**Table 4:** eigenvalues and variance explained by the principal component analysis

Components	Initial eigenvalues		
	Total	% of Variance	Cumulative %
1	4.996	24.978	24.978
2	1.714	8.568	33.545
3	1.648	8.241	41.786
4	1.401	7.006	48.792
5	1.270	6.349	55.141
6	1.044	5.218	60.360
7	1.001	5.006	65.366
8	948	4.738	70.104
9	835	4.177	74.281
10	724	3.619	77.900
11	673	3.366	81.266
12	656	3.280	84.546
13	603	3.016	87.562
14	501	2.505	90.067
15	475	2.373	92.440
16	377	1.883	94.323
17	365	1.827	96.150
18	332	1.659	97.809
19	225	1.126	98.935
20	213	1.065	100.000
**Rotation sums of squared loadings**			
1	**Total**	**% of Variance**	**Cumulative %**
2	2.687	13.437	13.437
3	2.367	11.837	25.274
4	2.130	10.652	35.927
5	1.786	8.932	44.859
6	1.602	8.011	52.869
7	1.354	6.771	59.641
8	1.145	5.725	65.366

**Factor structure, naming and loading:**the rotated component matrix in Table 5 presents the factor structure, coefficients of loadings, their eigenvalue, percent of variance, and a common name that could describe each factor. In this study, six-factor structures of social interaction impairments were identified: factor 1 challenges in interpersonal relationship and affection (eigenvalue: 4.99, variance: 13.437%), factor 2. emotional insensitivity (eigenvalue: 1.714, variance: 11.837), factor 3 poor gesture and attention (eigenvalue: 1.648, variance: 10.652), factor 4 social withdrawal (eigenvalue: 1.40, variance: 8.932), factor 5 behavioral resistance (eigenvalue: 1.270, variance: 8.011), and factor 6 limited in sharing (eigenvalue: 1.044, variance: 6.771). These factors accounted for about 60% of the total variance.

**Table 5:** rotated component matrix from a principal component analysis with orthogonal varimax rotation

Items	Factors						Communalities
	1	2	3	4	5	6	
	Challenges in interpersonal relationships and affection	Emotional insensitivity	Poor gesture and attention	Social withdrawal	Behavioral resistance	Limited in sharing	
8. Fails to greet parents	**.810**	032	185	034	-078	-002	714
7. Show no affection	**.774**	-.082	.160	.189	-009	-053	717
16. Lack of friends/companions	**.610**	.237	.084	.176	120	-012	520
9. Avoid contact with others	**.521**	201	-065	192	151	324	509
1. Not sociable/approachable	**.396**	332	.255	.290	048	-386	568
19. Indifferent to being liked	074	**.870**	140	075	-043	005	791
20. Indifferent if parent(s) leave	075	**.848**	029	085	092	043	743
18. Insensitive to others' feelings	096	**.645**	126	153	-033	113	791
13. Do not wave bye-bye'	060	093	**.733**	081	072	048	578
10. Does not imitate	099	047	**.728**	369	014	028	683
5. No eye contact	215	082	**.683**	018	-158	108	663
3. Pay little or no attention when addressed	341	298	**.492**	051	195	-099	499
2. Ignore other people	127	299	112	**.753**	263	-117	767
6. Prefer to be left alone	.294	074	224	**.722**	-022	030	674
17. Does not smile	008	080	218	**.487**	050	487	607
15. Temper tantrums	-112	-010	056	198	**.774**	-120	676
4. Uncooperative and resistant	180	-017	-079	030	**.708**	335	655
14. Disagreeable/not compliant	532	186	236	-195	**.537**	079	730
12. Does not share or show	021	093	.081	-053	078	**.818**	702
11. Dislikes being held/cuddled	063	098	-019	096	.099	-068	734
Eigenvalue	4.996	1.714	1.648	1.401	1.270	1.044	
Explained Variance %	13.437	11.837	10.652	8.932	8.011	6.771	
Cumulative%	13.437	25.274	35.927	44.859	52.869	59.641	

The top six behavioral patterns of social interaction impairments are labeled 1 through 6, each representing a distinct underlying structure. Displayed in bold shows the significant variables associated with each pattern.

Items 8 (Fails to greet parents), 7 (Show no affection), 16 (Lacks friends/companions), 9 (Avoid contact with others), and 1 (Not sociable/approachable) loaded more strongly to factor 1. Items 19 (Indifferent to being liked), 20 (Indifferent if parent(s) leave), and 18 (Insensitive to others' feelings) loaded to factor 2. In factor 3, four correlated items were identified: 13 (Does not wave bye-bye), 10 (Does not imitate), 5 (No eye contact), and 3 (Pays little or no attention when addressed) loaded to factor 3. The fourth factor included three items: 2 (Ignore other people), 6 (Prefer to be left alone), and 17 (Does not smile). “Items 15 (Temper tantrums), 4 (Uncooperative and resistant), and 14 (Disagreeable/not compliant) were significantly loaded onto factor 5, while item 12 (Does not share or show) was significantly loaded onto factor 6.

## Discussion

PCA is a multivariate statistical method that is widely used in computational genetics to examine numerous variables simultaneously [36,37]. In genetics, sets of genes expressed at the same time and regulated by a shared transcription factor or regulatory network [38-40] are called co-expressed genes, and they are involved in related biological processes or pathways. Similarly, different functional impairments in autism that manifest concurrently are likely governed by a related regulatory mechanism, offering insights into how different behaviors may be interconnected [41,42]. For instance, different behavioral traits associated with social interaction may arise from shared transcription factors or underlying biological mechanisms. PCA with orthogonal varimax rotation and related statistical methods can be used to understand the patterns of different behavioral features in ASD [43]. These approaches can be applied to data from various standardized diagnostic instruments for ASD: questionnaires, parent interviews, and observation schedules.

Using ATEC as a questionnaire tool of parent interview and the varimax rotation method, our study revealed six-factor structures of social interaction impairments in children diagnosed with ASD, and the factors identified include difficulties in interpersonal relationships, emotional insensitivity, poor gestural communication, limited attention, social withdrawal, behavioral resistance, and restricted sharing of interests or experiences. These multiple-factor structures indicate that social interaction impairments are constructed of different latent variables, and can be categorized into distinct dimensions [33,43].

This result is in line with the existing literature that social interaction impairments in ASD are made from different and hidden variables, which can be categorized into distinct dimensions. Using the ATEC, Abaoud *et al*. [6] found a two-factor structure in the sociability domain. Similarly, Gau *et al*. [43], using the social communication questionnaire (SCQ), found three-factor solutions including social interaction, repetitive behaviors, and communication. Furthermore, our findings are consistent with the study of Georgiades *et al*. [45], who identified five-factor structures in social interaction among children with ASD, strengthening the idea that social functioning in ASD is a multidimensional construct. Variability in the number of factor structures across studies may reflect differences in the cultural context, measurement tools, and sample characteristics. In our study, social and environmental factors may influence the expression of ASD symptoms, potentially contributing to additional dimensions.

In the present study, the variance explained by the factors was also calculated, and it accounted for approximately 60% of the variance. All items showed high standard loading, ranging from 0.396 to 0.870. This result was exactly similar to the study by Netson *et al*. [46], which reported that the sociability domain of the ATEC accounted for 60% of the variance, with all items showing high standard loadings above 0.3. The similarity may indicate the importance of ATEC in understanding the social deficits associated with ASD, making it a valuable tool for identifying specific areas of difficulty and tailoring interventions accordingly. The strong factor loadings in both studies also indicate that all items in the sociability domain contribute meaningfully to the measurement of social interaction impairments of the study subjects.

**Limitations:** this study has presented scientific evidence on the underlying factor structure of social interaction impairments in children diagnosed with ASD at autism centers in Addis Ababa, Ethiopia. However, there were certain limitations. The first limitation was that the study was conducted only with autistic children found in autism centers and did not include autistic children found in the community. Second, it was carried out only in cases, and it was difficult to infer the underlying mechanism that contributed to the observed outcome. Thus, it was recommended to conduct a study on the factor structure of sensory and cognitive impairments among children diagnosed with ASD who are found in the community, and a case-control study to assess the risk factors of autism.

## Conclusion

This study demonstrated six different patterns of social interaction impairments in children diagnosed with ASD, including challenges in interpersonal relationships, emotional insensitivity, ineffective gestural communication, diminished attention, social withdrawal, behavioral resistance, and limited sharing of interests or experiences. It is important to consider these patterns when supporting children with autism spectrum disorder.

### What is known about this topic

- Previous literature has explored limited and unlabeled factor structures within the sociability domain of ASD, utilizing principal component analysis of the autism treatment evaluation checklists.

### What this study adds

- This study identifies a broader dimension of the factor structure of social interaction impairments, and each factor is labeled based on its shared conceptual meanings;
- The study also provides the variance explained by the identified factors, providing a more comprehensive understanding of the underlying constructs.
